# Rare earth element identification and quantification in millimetre-sized Ryugu rock fragments from the Hayabusa2 space mission

**DOI:** 10.1186/s40623-022-01705-3

**Published:** 2022-09-28

**Authors:** Pieter Tack, Ella De Pauw, Beverley Tkalcec, Miles Lindner, Benjamin Bazi, Bart Vekemans, Frank Brenker, Marco Di Michiel, Masayuki Uesugi, Hisayoshi Yurimoto, Tomoki Nakamura, Kana Amano, Megumi Matsumoto, Yuri Fujioka, Yuma Enokido, Daisuke Nakashima, Takaaki Noguchi, Ryuji Okazaki, Hikaru Yabuta, Hiroshi Naraoka, Kanako Sakamoto, Shogo Tachibana, Toru Yada, Masahiro Nishimura, Aiko Nakato, Akiko Miyazaki, Kasumi Yogata, Masanao Abe, Tatsuaki Okada, Tomohiro Usui, Makoto Yoshikawa, Takanao Saiki, Satoshi Tanaka, Fuyuto Terui, Satoru Nakazawa, Sei-Ichiro Watanabe, Yuichi Tsuda, Laszlo Vincze

**Affiliations:** 1 Dept. of Chemistry, XMI, Ghent University, Krijgslaan 281 S12, 9000 Ghent, Belgium; 2 Dept. of Geoscience, Goethe University, Altenhoeferallee 1, 60438 Frankfurt, Germany; 3 IHGP, University of Hawaii, Menoa, HI USA; 4 The European Synchrotron, ESRF, 38000 Grenoble, France; 5 JASRI/SPring-8, Sayo, 679-5198 Japan; 6 Hokkaido University, Sapporo, 060-0810 Japan; 7 Tohoku University, Sendai, 980-8578 Japan; 8 Department of Earth Science, Tohoku University, Aoba-ku, Sendai, 980-8578 Japan; 9 Kyoto University, Kyoto, 606-8502 Japan; 10 Kyushu University, Fukuoka, 812-8581 Japan; 11 Hiroshima University, Higashi-Hiroshima, 739-8526 Japan; 12 ISAS/JAXA, Sagamihara, 252-5210 Japan; 13 The University of Tokyo, Tokyo, 113-0033 Japan; 14 Institute of Space and Astronautical Science (ISAS), Japan Aerospace Exploration Agency (JAXA), Sagamihara, 252-5210 Japan; 15 Kanagawa Institute of Technology, Atsugi, 243-0292 Japan; 16 Nagoya University, Nagoya, 464-8601 Japan

**Keywords:** Fundamental parameter quantification, Hayabusa2, REE, Ryugu, X-ray fluorescence spectroscopy

## Abstract

Millimetre-sized primordial rock fragments originating from asteroid Ryugu were investigated using high energy X-ray fluorescence spectroscopy, providing 2D and 3D elemental distribution and quantitative composition information on the microscopic level. Samples were collected in two phases from two sites on asteroid Ryugu and safely returned to Earth by JAXA’s asteroid explorer Hayabusa2, during which time the collected material was stored and maintained free from terrestrial influences, including exposure to Earth’s atmosphere. Several grains of interest were identified and further characterised to obtain quantitative information on the rare earth element (REE) content within said grains, following a reference-based and computed-tomography-assisted fundamental parameters quantification approach. Several orders of magnitude REE enrichments compared to the mean CI chondrite composition were found within grains that could be identified as apatite phase. Small enrichment of LREE was found for dolomite grains and slight enrichment or depletion for the general matrices within the Ryugu rock fragments A0055 and C0076, respectively.

**Graphical Abstract:**

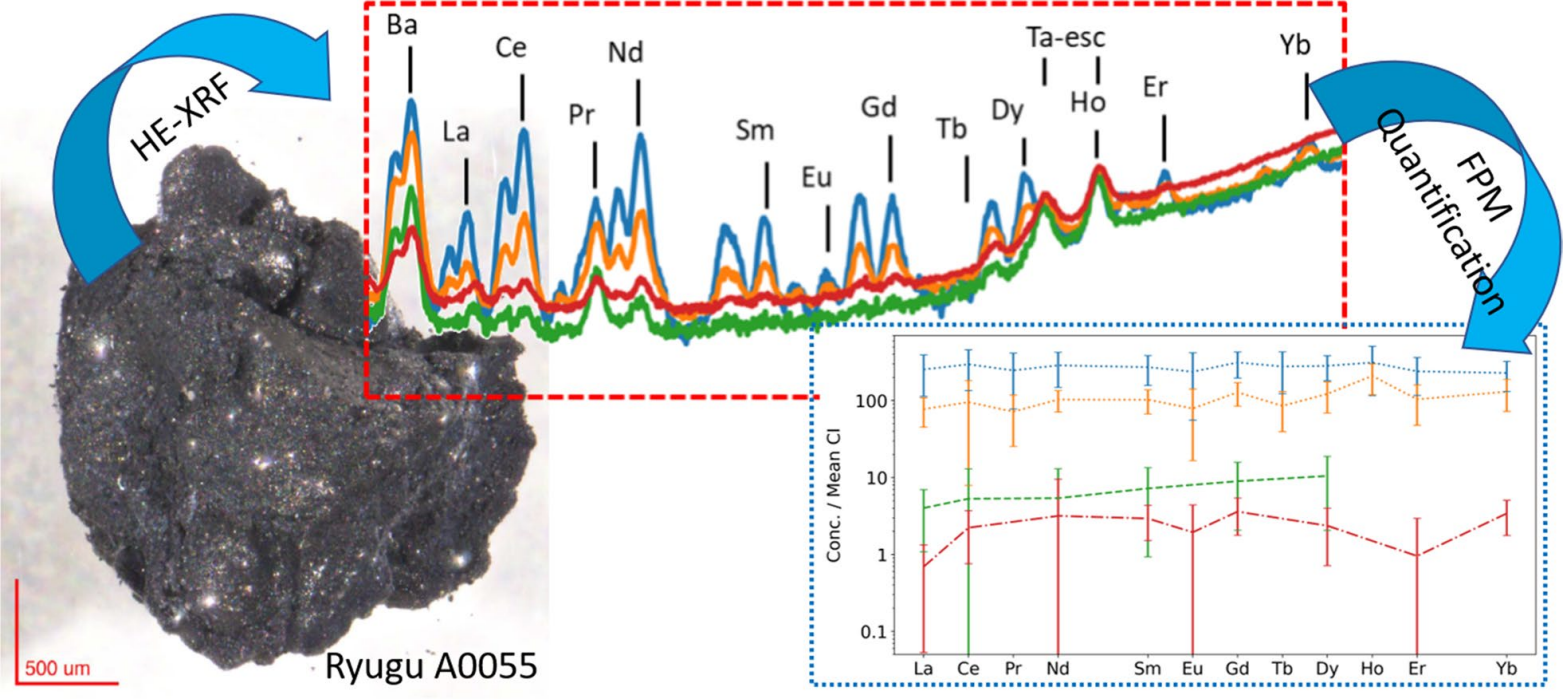

**Supplementary Information:**

The online version contains supplementary material available at 10.1186/s40623-022-01705-3.

## Introduction

As meteoroids enter Earth’s atmosphere they are decelerated and are exposed to frictional heat, potentially resulting in the chemical alteration of their composition by evaporation of (trace amounts of) volatile elements and dehydration (Rudraswami et al. 2015; Greshake et al. 1994). Furthermore, after impacting on Earth’s surface they are exposed to various terrestrial alteration processes. In contrast, the samples investigated in this work were collected directly from the surface and subsurface regions of asteroid Ryugu during two surface touchdowns by the Hayabusa2 space mission of the Japanese Aerospace Exploration Agency (JAXA) and were kept in pristine conditions during its return to Earth (Tachibana et al. 2014; Watanabe et al. 2017). Therefore, any evidence for alteration in the Ryugu material can be assumed to have occurred in situ on the asteroid, a carbon-rich carbonaceous asteroid type Cb, whose surface material is thought to be similar to CI or CM carbonaceous chondrites (Hamilton et al. 2019; Kitazato et al. 2019). Such evidence includes the presence of secondary phases, such as phyllosilicates (predominantly serpentine and saponite), magnetite, sulphides, and carbonates (Yokoyama et al. 2022), which have often been observed also in other CI and CM chondrites (Tomeoka and Buseck 1988; Gounelle and Zolensky 2014; Lee and Nicholson 2009; King et al. 2015; Bates et al. 2019; Tkalcec et al. 2022). During the return and curation of the samples they remained within a protective environment and free from Earth’s atmosphere’s influences ready for subsequent analysis.

As such, the investigation of these samples by non-destructive and non-invasive methods first is of the utmost importance, to obtain as much information as possible while minimising any form of alteration of the sample during these initial investigations. For this purpose, hard X-ray-based analysis methods are a prime candidate due to their highly penetrating yet fully non-invasive character.

In this study, two rock fragments, A0055 and C0076, from the surface and sub-surface sampling procedures from Ryugu (Sawada et al. 2017), were investigated by high energy synchrotron radiation X-ray fluorescence (HE-SR-XRF) spectroscopy at the “materials chemistry and materials engineering beamline” ID15A of the European Synchrotron Radiation Facility (ESRF, Grenoble, France) (Vaughan et al. 2020; De Pauw et al. 2022). The goal was to obtain elemental distribution maps of the millimetre-sized samples to identify potential regions of interest for further (invasive) analysis. In what follows, a detailed description of the performed analysis is provided, along with a quantification approach to obtain rare earth element (REE) trace level concentration values from mineral grains even at significant depth within the sample matrix (> 500 µm).

The obtained REE concentrations are normalised to the mean CI chondritic REE composition (Lodders 2021) and compared to apatite in the CI-chondrite Orgueil and in other carbonaceous chondrites.

## Materials and methods

### Sample description

Ryugu rock fragments A0055 and C0076, collected in chambers A and C, respectively, during the first and second touchdown on Ryugu’s surface by the JAXA Hayabusa2 sample return mission, were investigated using high energy XRF spectroscopy. Rock fragment A0055 measures 2.38 × 1.94 × 1.69 mm^3^, whereas rock fragment C0076 measures 2.53 × 1.74 × 1.41 mm^3^. Both samples were mounted on a 500 µm thick carbon pad on top of a 3 mm diameter aluminium cylinder. Before, during and after SR-XRF analysis, the sample was sealed within a 20 µm thin polyimide foil cap, secluding it from Earth’s atmosphere. In addition, before and after the SR-XRF experiments the polyimide-encapsuled samples were stored in air- and moisture-free conditions to maintain the samples’ pristine conditions during transport on Earth.

A total of 4 standard reference materials were used for the XRF quantification: an NIST SRM 611 (Trace elements in glass, ~ 500 ppm level trace element concentrations, thickness 1 mm), a NIST SRM 613 (Trace elements in glass, ~ 50 ppm trace element concentrations, thickness 1 mm and 100 µm), and an MPI-DING Atho-G reference material (rhyolitic glass geological reference material, thickness 100 µm) (Jochum et al. 2000, 2011).

### Synchrotron radiation X-ray fluorescence (SR-XRF) spectroscopy

**Fig. 1:**
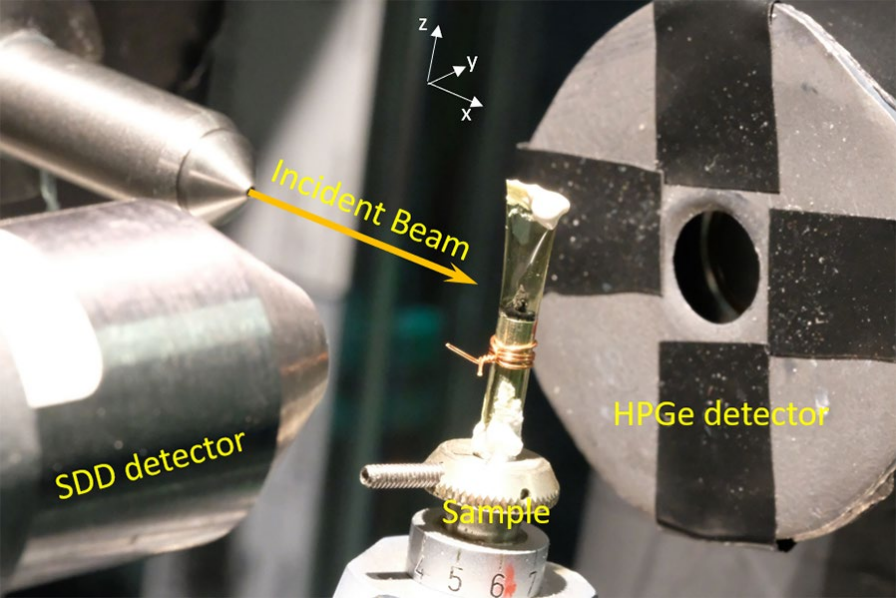
Instrumental overview photograph of the setup as applied at beamline ID15A (ESRF, Grenoble, France) during the experiment

Experiments were performed at the ID15A beamline of the ESRF (Grenoble, France) using a setup similar to that used during preparatory research for the Hayabusa2 return sample preliminary analysis (De Pauw et al. 2022) (Fig. 1). A 90 keV beam with focal spot size of approximately 0.5 × 0.5 µm^2^ at the sample position was obtained with a total flux of approximately 10^11^ photons/s. A Canberra Mirion Cryo-pulse 5plus HPGe detector was used to detect the emitted X-ray photons, mounted at a 90° angle with respect to the incident photon beam in the plane of polarization to minimize Compton scatter contribution, at the left side of the sample. An In and Ta containing detector collimator was implemented to remove parasitic scattering from Kirkpatrick–Baez focussing mirrors, air, guard slits and beam monitor PIN diode. A Pb detector collimator was used to prevent spectral contribution from the instrument surrounding the sample environment (sample holder and sample alignment stages).

XRF spectra were integrated using the PyMca5 software package (Solé et al. 2007) and further processed for quantification as described in the results and discussion section of this manuscript. All data were corrected for primary beam flux and detector dead time. Mean CI chondritic composition concentrations were obtained from Lodders et al*. (*Lodders 2021*)*, completed by oxygen to obtain a total 100 wt% matrix representation. A bulk Ryugu sample density of 1.81 g/cm^3^ was assumed, which is similar to findings by Nakamura et al*. (*Nakamura et al. 2022b)*.*

Computed tomography (CT) data were processed using the TomoPy ‘gridrec’ reconstruction algorithm (Gursoy et al. 2014) and were compared to XRF–CT data to obtain information on particular features of interest. Particular attention was given to local Ca-, REE-, Fe- or S-enrichments, for example, in conjunction with the morphology, absorption characteristics and CT grayscale tone of the associated phases.

## Results and discussion

**Fig. 2:**
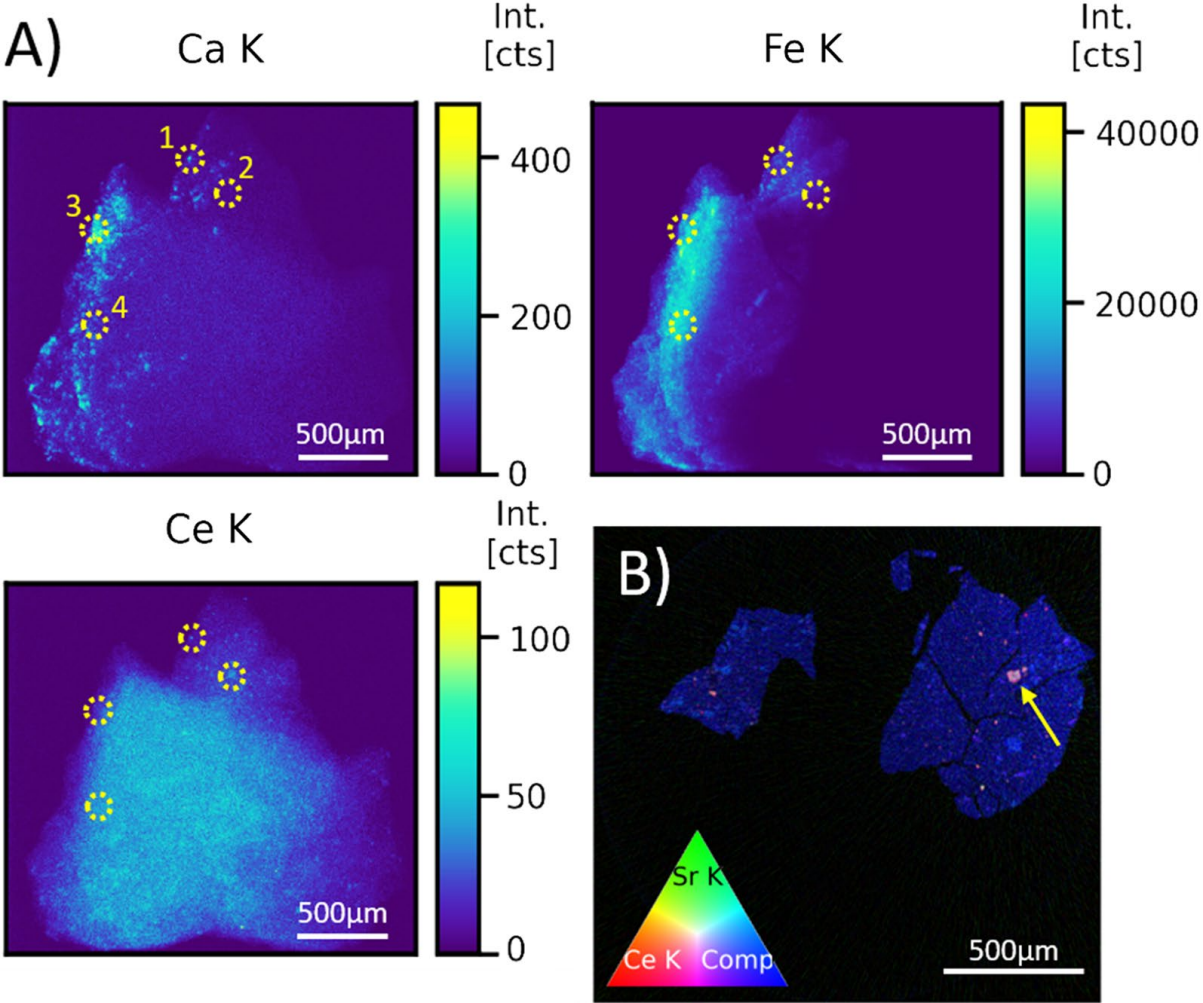
XRF overview elemental distribution images (5 µm step size, 0.2 s/pt) for Hayabusa2 return sample A0055 (**A**) and XRF–CT cross-sectional RGB image (**B**) presenting a virtual horizontal cut through point 2 displaying the Ce, Sr and Compton scatter distribution within the sample (5 µm × 0.4° steps, 0.2 s/pt). Yellow marks and numbers indicate the points of interest that were further investigated

An initial overview scan was made of each sample to identify the REE distribution in the sample, as displayed in Fig. 2. It should be noted that this overview scan is in essence a 2D projection of the 3D sample, where the sample depth from which information is projected on the 2D image is dependent on the monitored fluorescence line energy, the sample composition/topology and density along both the projection path (i.e., along the incident beam) as well as the corresponding escape path of the fluorescence signal toward the detector. For the low-Z elements this corresponds to a few tens of micrometre (e.g., < 100 µm for Ca–K_α_), whereas for the higher Z elements this increases to several millimetre (e.g., < 1.5 cm for La–K_α_), limited by the actual sample thickness at the given primary X-ray beam position. In addition, as the detector is located on the left side of the sample, this side is characterised by more intense signal as the resulting radiation is less influenced by self-absorption effects within the sample. Procedures to correct for these self-absorption effects exist but are often arduous in nature and demand a high degree of knowledge on the sample’s internal and external morphology and composition. Furthermore, these methods are often characterised by a large degree of uncertainty, especially when correcting low intensity pixels, such as the pixels on the right side of the image, Fig. 2A, due to the large uncertainties governed by Poisson statistics and insufficient knowledge on the absorption coefficients and density distribution along the excitation and detection path within the highly heterogeneous sample material. As such, the images presented here are not self-absorption-corrected and provide an overview similar to what can typically be readily obtained during a synchrotron radiation experiment with limited to no post-processing.

The elemental distribution images for A0055 shown in Fig. 2A display a large amount of calcium-rich grains, some of which correlate with an enhanced REE signal represented here by the Ce distribution image, the latter being the most abundantly present REE in CI chondrites (Lodders 2021) and selected here to represent all detected REEs. The iron distribution image displays a generally Fe-rich background, superimposed by occasional platelet or needle-like Fe-enhanced structures. Several points of interest were identified for further investigation, marked in Fig. 2A by yellow circles. Points 1 and 2 are both REE-rich, and Point 1 is also visibly enriched in Ca. This is likely also the case for point 2, but as the signal from point 2 arises from deeper (measured along the detector axis) within the sample bulk, the emitted fluorescence radiation must pass through a larger fraction of the sample before reaching the detector, which has a more negative effect on the Ca detection but less so for the REEs due to their high K-line photon energies and associated larger information depth. Point 3 is also characterised by a high Ca signal, yet unlike point 1 does not display significant REE enrichment. Point 4 is additionally selected as a good representative for the general sample matrix, as it displays no particular enrichment in any of the detectable elements.

An XRF–CT scan was acquired through point 2 (Fig. 2B), displaying the Sr and Ce distribution within the sample, as well as the Compton signal which provides a general measure for the sample density and morphology. It is clear from this image that the REE-rich region that is identified in Fig. 2A (point 2) can be attributed to an approximately 80 µm large grain, displayed in the RGB image as a yellow–white grain indicated by a yellow arrow, containing increased levels of Sr and REEs compared to the sample matrix. It was previously reported that there is a strong correlation between the Ca and Sr distribution (Tkalcec et al. 2022), thus allowing for the inference that this grain of interest is enriched not only in REEs and Sr, but also in Ca, further suggesting that point 2 is chemically very similar to point 1.

The high energy (90 keV) beam at beamline ID15A is especially suited for the detection of heavier elements, such as REEs. Unfortunately, direct detection of light elements such as C, O, Mg, Si or P is not feasible at ID15A due to the strong absorption of their X-ray fluorescence photons by the sample surrounding air and the sample matrix. For this reason, identification of these grains by SR-XRF in such a mm-sized particle without prior invasive sample preparation, such as targeted cutting or polishing, must be achieved indirectly. First analyses of Ryugu material have reported that the main Ca-bearing phases are dolomite and apatite, and that anhydrous Ca-bearing silicate phases such as pyroxene or anorthite are rare (Nakamura et al. 2022a). Given the small grain size of points 1 and 2 and the typical enrichment of REEs within Ca-phosphate phases (Morlok et al. 2006), it is suggested that the joint Ca and REE signals from points 1 and 2 both originate from apatite phases. This is in agreement with recently reported REE concentrations in several other Ryugu samples that confirm the highest REE enrichments are found in apatite (Yokoyama et al. 2022).

Unlike points 1 and 2, the lack of distinctly visible REE fluorescence signal from point 3 together with its much larger grain size (Fig. 2A, top left image) suggests that point 3 is not an apatite grain, despite its strong Ca-rich signal.

**Fig. 3:**
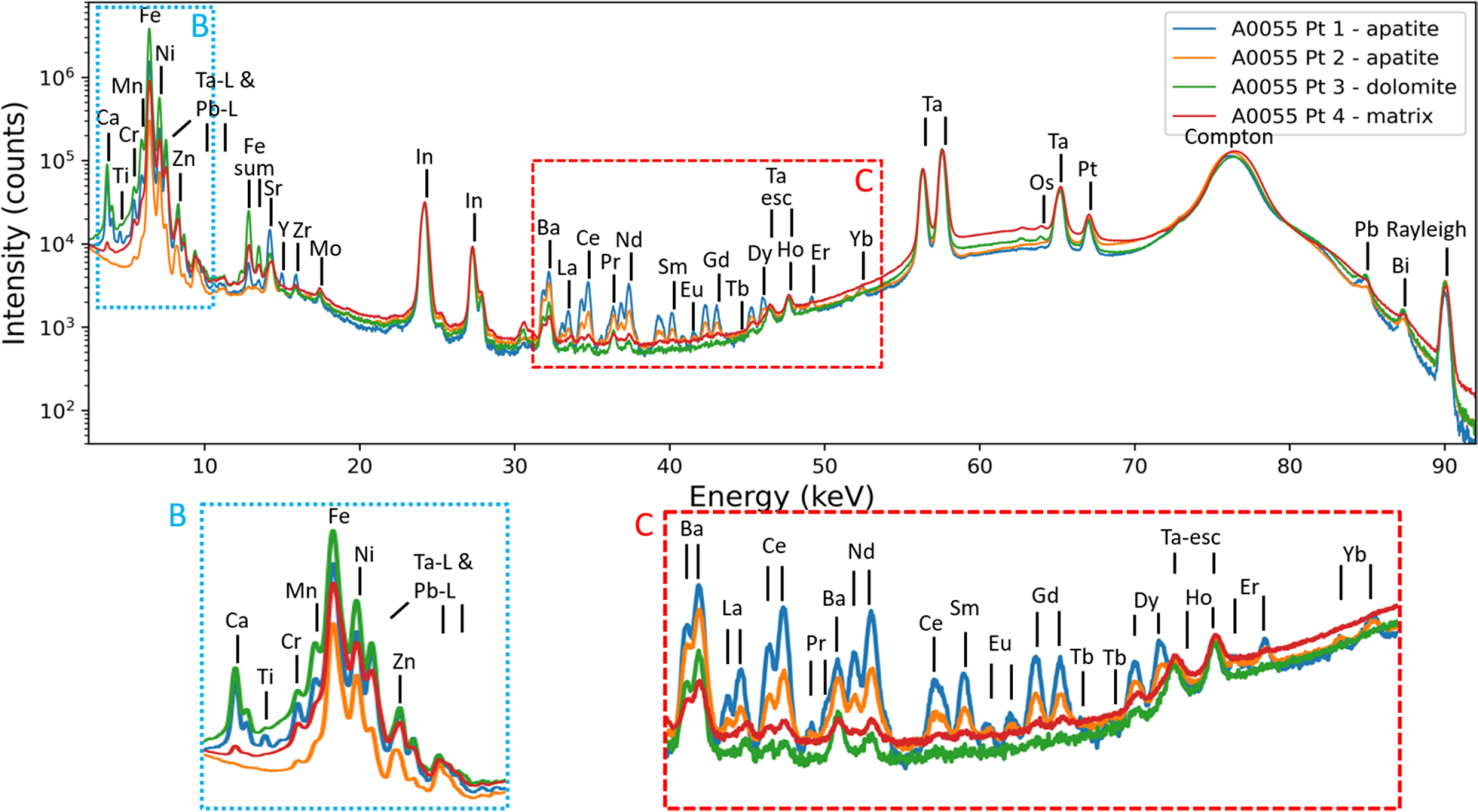
XRF spectra corresponding to the measurements in points 1 to 4 in rock fragment A0055, as indicated in Fig. 2A (600 s/pt). A clear enhancement in the REE region is observed for points 1 and 2. XRF spectra were normalised for the Ta–K_α_ signal intensity to provide more straightforward comparison. Magnified inserts of two select energy ranges marked by dashed bounding boxes in light blue and red are displayed in parts B and C, respectively

An identical scanning procedure as described above was followed for rock fragment C0076. For quantitative purposes, measurements with longer acquisition time (600 s) compared to the overview scans were performed in both samples, A0055 and C0076, to acquire better counting statistics and signal-to-noise ratios. The local analyses were performed on the four points indicated in Fig. 2A as well as on four points within C0076 identified as corresponding to an apatite grain and three points corresponding to sample matrix. The resulting XRF spectra for A0055 points 1–4 are displayed in Fig. 3 (and Additional file 1: Fig. S1 in the Additional File for C0076 XRF spectra of points 1–4). It should be noted that the Ta and In signals originate from the used detector collimator, and as such scale with the Rayleigh and Compton intensities and are not to be attributed to the sample composition. All spectra correspond to photons detected from down to a given sample depth along the excitation and detection beam path, limited by the photon-energy-dependent information depth. Given the particle’s topology, lower energy photons will only contribute to the spectrum from a relatively short segment along the beam path, whereas higher energy photons (REEs and higher atomic-number elements) effectively contribute from the full sample thickness. Nevertheless, a clear distinction can be made between the different points of interest based on the REE contribution to the separate spectra.

The spectral results shown in Fig. 3 confirm that Ryugu fragment A0055 points 1 and 2 are enriched in REEs compared to points 3 and 4. Furthermore, almost all REEs are detected: La, Ca, Pr, Nd, Sm, Eu, Gd, Tb, Dy, Ho, Er, Yb and Lu. A note should be made for Ho, as this signal displays spectral overlap with the Ta escape peaks, which are abundantly present in all measurements due to the considerable Ta and In collimator signal contribution. However, the applied spectral deconvolution software takes these escape peak ratios into account, thus providing a reliable integrated signal for Ho as well for all but the noisiest spectra. In addition, points 1 and 3 display a strong Ca signal. As discussed previously, the Ca signal originating from point 2 is absorbed by the rest of the sample bulk hindering its detection under the measurement geometry that was applied during the acquisition of the images displayed in Figs. 2A and 3. The spectral results of A0055 further confirm that point 3 of A0055 has a different mineralogy to points 1 and 2. In addition to its weaker REE signal, a clear Mn-enrichment is observed in A0055 point 3 (Fig. 3B) which, together with its larger grain size relative to points 1 and 2, supports its identification as a dolomite grain. This is in agreement with first analyses of other Ryugu samples that have reported a widespread abundance of coarse-grained (~ 100 µm) dolomite that also contains a rhodochrosite (MnCO_3_) component of ~ 9 mol% (Nakamura et al. 2022a), and is further in line with reports of carbonate grains in other CI and CM carbonaceous chondrites, where the concentration of Mn is known to be significantly higher in dolomite relative to calcite or aragonite (Lee et al. 2014; Riciputi et al. 1994).

### Quantitative analysis of point spectra

A reference material-based fundamental parameter quantification approach was used to obtain quantitative data corresponding to the point measurement data. It can be shown that the fluorescent line intensity of element *i*, I_i_, can be calculated following Eq. (1) (Schoonjans 2012; Schoonjans et al. 2012; Szalóki et al. 2017). Here, I_0_ represents the primary photon beam flux impinging the sample, *w*_*i*_ is the weight fraction of element *i* in the sample, Q_i_ is the so-called XRF production cross section for the XRF line of interest for element *i*, G is the detector solid angle and efficiency factor (geometry factor), ρ and T the sample density and local thickness values, respectively. χ represents a measurement geometry and sample composition dependent attenuation coefficient combining the sample’s mass attenuation coefficients at the incident and fluorescence line energies, respectively. Using χ, the last term in the equation, $\left(\frac{1-{e}^{-\chi \rho \mathrm{T}}}{\chi \rho \mathrm{T}}\right)$, can be interpreted as an absorption correction term representing the influence of sample matrix effects. In case of the high energy XRF spectral data presented below, this factor is close to 1 due to the limited absorption of the high energy REE K_α_ emission lines by the Ryugu sample matrix (e.g., the La–K_α_ attenuation length within a 1.81 g/cm^3^ CI matrix is approximately 3.3 mm):

1 $${\mathrm{I}}_{\mathrm{i}}= {\mathrm{I}}_{0}\mathrm{G}{w}_{i}{\mathrm{Q}}_{\mathrm{i}}\rho \mathrm{T}\left(\frac{1-{e}^{-\chi \rho \mathrm{T}}}{\chi \rho \mathrm{T}}\right)$$

2 $${\mathrm{Y}}_{\mathrm{i}}= \frac{{\mathrm{I}}_{\mathrm{i}}}{{w}_{i}\rho \mathrm{T}}$$

**Fig. 4:**
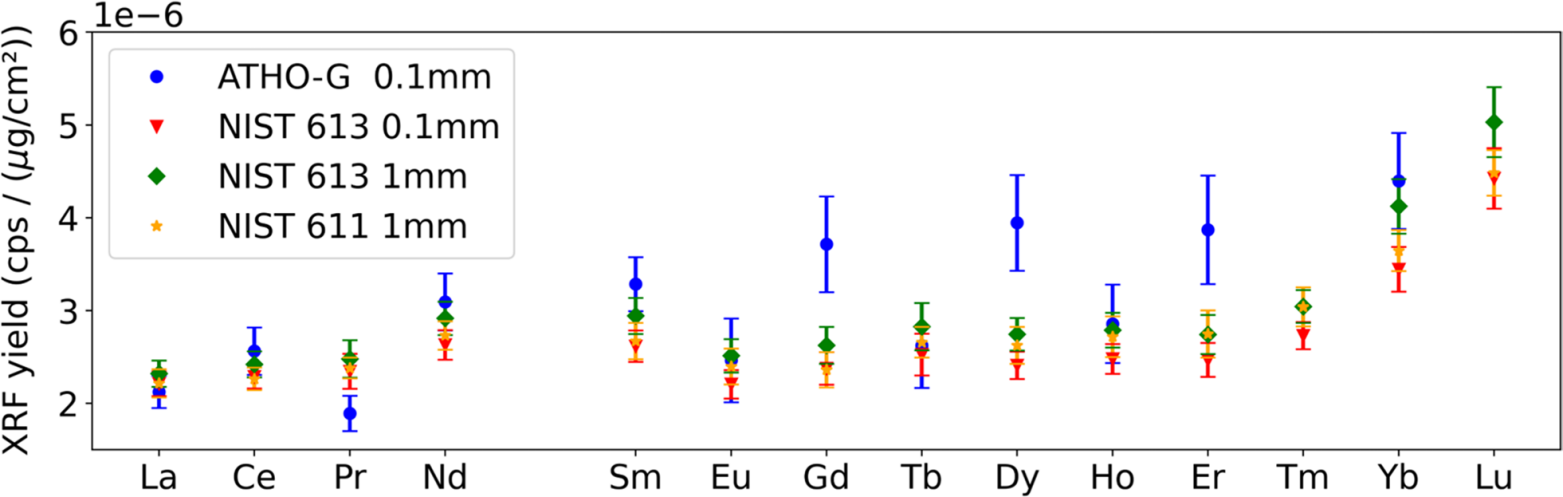
XRF yields for the different reference materials that were investigated, as calculated by Eq. 2. Error bars indicate three standard error margins (99.7% confidence interval)

Several terms in Eq. (1) are identical when comparing a reference material to an unknown sample, measured under the same experimental conditions: I_0_ (after appropriate normalisation which also takes into account any acquisition time differences), G and Q_i_. In addition, also the absorption correction term is considered equal for the used reference materials and the unknown sample, due to the relatively high REE K-line photon energies and the limited absorption of these photons by the sample matrices. These simplifications allow us to define an “XRF yield” expressed by Eq. (2) (Fig. 4). It is clear that for all 4 reference materials very similar XRF yields are obtained, with minor deviations in the case of MPI-DING Atho-G for certain elements: a clear indication that the density and sample thickness, as well as the matrix absorption effects, are appropriately taken into account for the REE range. For further data evaluation, the average value of the different yields for a given element were used, with a corresponding error equal to the standard deviation of the average or derived from standard error propagation, whichever error gave the largest value.

**Fig. 5:**
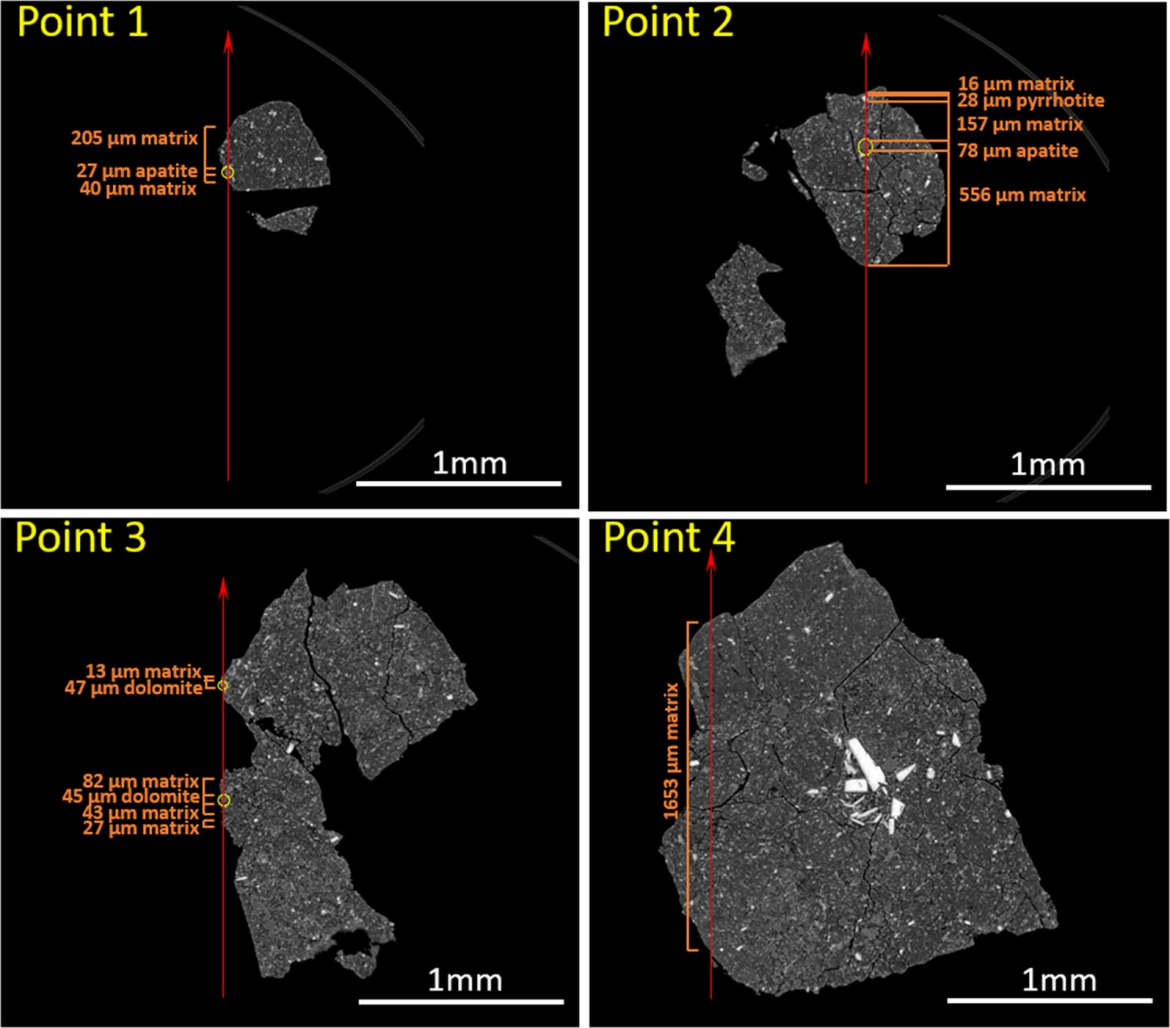
CT slices that were obtained at SPring-8 beamline 20XU (Nakamura et al. 2022b) show the positions of the points of interest indicated in Fig. 2A. A red arrow marks the primary X-ray beam path and direction, fluorescence detector was positioned at the left of the CT image. Yellow circles indicate the Ca-rich grains (points 1–3) from which REE information is primarily obtained. Point 4 is a matrix measurement and as such has no distinct Ca-rich grains. Separate grains along the beam path are indicated by their respective size along the beam path in orange, along with an estimate of the mineralogical phase for the larger grains

Equation (2) can now be used to quantify the data obtained from the unknown samples, provided that the sample local density and thickness are known. Due to the heterogeneous nature of the samples, however, this is not a trivial piece of information to obtain. For this purpose, CT scans acquired at the sample heights corresponding to the point measurements (Fig. 5) were used. For each point measurement, the primary beam path was divided into a number of segments based on the presence of grains along the path. Where possible, mineral phases along the beam path were identified, and their corresponding density was taken into account. A mean matrix density of 1.81 g/cm^3^ was used (Nakamura et al. 2022b). A self-absorption correction was performed to account for the REE signal absorption within the sample, between the point of excitation and ultimately reaching the detector, by comparing the experimental Fe–K_α_ over Fe–K_β_ signal intensity ratio to the theoretical radiative rate ratio (De Samber et al. 2010; Trojek et al. 2008). The difference between experimentally obtained and theoretical ratio is contributed to absorption by the sample matrix, for which a corresponding absorption path length was calculated following Lambert–Beer’s law ($I={I}_{0}{e}^{-\mu \rho T}$). This absorption layer was then used to correct the experimentally obtained REE signal intensities. Using the values stated in Fig. 5, an average (feature size weighted) density and total path length can be determined, which are then used as the respective *ρ* and *T* values in Eq. (2) to calculate the average REE concentration within the full path length detected through the sample as indicated in Fig. 5.

**Fig. 6:**
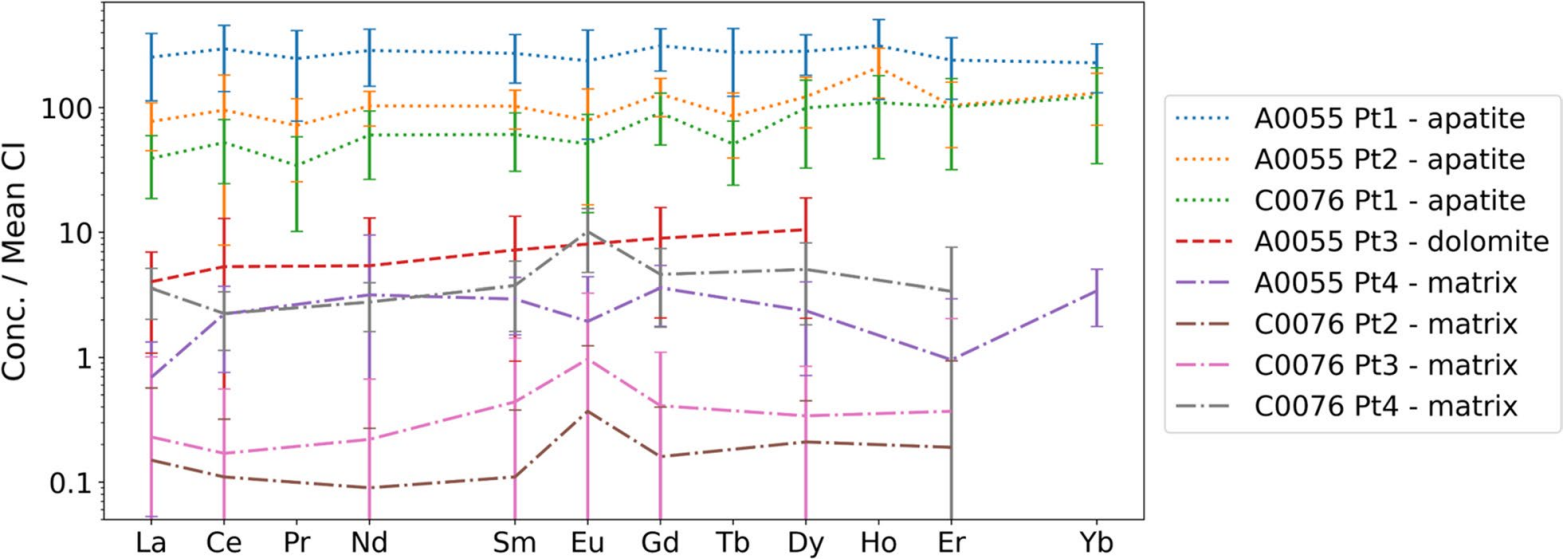
REE trend of different phases within the Ryugu A0055 and C0076 rock fragments. Error bars indicate three standard error margins (99.7% confidence interval)

However, the REEs are not distributed evenly throughout the full beam path, but are expected to be concentrated within the Ca-phosphate-rich phases along the beam path (Morlok et al. 2006). As a result, the REE information contained within the spectra in Fig. 3 and Additional file 1: Fig. S1 does not correspond to the full sample thickness but instead originates mainly from a smaller grain along this beam path. This is done by calculating the concentration values following Eq. (2), but using the *ρ* and *T* values corresponding to the grain of interest instead of an average density and total intersected sample path length. The obtained concentration values can then be directly compared to the mean CI chondritic composition (Lodders 2021), as displayed in Table 1 and Fig. 6.

**Table 1:** Rare earth element concentrations for the separate points of interest, compared to the reported mean CI chondrite (Lodders 2021) concentration

		Mean CI	Point 1	Point 2	Point 3	Point 4
A0055	La	0.24	61.35 (11.30)	18.71 (2.60)	0.97 (0.24)	0.17 (0.05)
	Ce	0.63	183.83 (33.42)	59.25 (6.04)	3.29 (1.58)	1.38 (0.30)
	Pr	0.10	23.23 (5.29)	6.75 (1.45)	–	–
	Nd	0.47	136.27 (21.89)	48.94 (5.07)	2.56 (1.21)	1.50 (0.33)
	Sm	0.15	41.86 (5.87)	15.79 (1.82)	1.11 (0.32)	0.45 (0.07)
	Eu	0.06	13.98 (3.57)	4.65 (1.22)	–	0.11 (0.05)
	Gd	0.21	64.84 (8.03)	26.55 (3.00)	1.85 (0.47)	0.75 (0.13)
	Tb	0.04	10.56 (1.96)	3.25 (0.58)	–	–
	Dy	0.25	72.52 (8.66)	31.30 (4.54)	2.69 (0.72)	0.61 (0.14)
	Ho	0.06	17.68 (3.70)	11.86 (1.70)	5.41 (0.99)	1.34 (0.28)
	Er	0.16	39.83 (6.82)	17.23 (3.10)	–	0.16 (0.11)
	Yb	0.17	38.62 (5.44)	22.06 (3.27)	–	0.58 (0.09)
C0076	La	0.24	9.46 (1.65)	0.04 (0.03)	0.05 (0.06)	0.86 (0.12)
	Ce	0.63	32.53 (5.75)	0.07 (0.04)	0.11 (0.08)	1.39 (0.23)
	Pr	0.10	3.23 (0.76)	-	–	–
	Nd	0.47	28.58 (5.32)	0.04 (0.03)	0.11 (0.07)	1.32 (0.19)
	Sm	0.15	9.37 (1.54)	0.02 (0.01)	0.07 (0.05)	0.58 (0.11)
	Eu	0.06	3.02 (0.72)	0.02 (0.02)	0.06 (0.05)	0.60 (0.11)
	Gd	0.21	18.76 (2.79)	0.03 (0.02)	0.08 (0.05)	0.95 (0.20)
	Tb	0.04	1.94 (0.34)	–	–	–
	Dy	0.25	25.46 (5.69)	0.05 (0.02)	0.09 (0.04)	1.29 (0.27)
	Ho	0.06	6.21 (1.34)	0.11 (0.03)	0.23 (0.06)	2.94 (0.47)
	Er	0.16	16.79 (3.84)	0.03 (0.04)	0.06 (0.09)	0.56 (0.23)
	Yb	0.17	20.62 (4.87)	–	–	–

All values are reported in ppm mass, values between brackets indicate the 1-sigma standard error, undetected elements are indicated by a dash (-)

The REE patterns for three apatite grains (points 1 and 2 in Ryugu fragment A0055 and point 1 in C0076, Fig. 6) all show enrichments of greater than approximately 30 times mean chondritic values. The highest enrichment is documented for point 1 in Ryugu fragment A0055 which shows REE enrichments of 250–300 times mean CI values and a noticeably flat pattern, indicating that this grain has not experienced significant fractionation-inducing processes on the asteroid Ryugu. The other two apatite spectra are less REE-enriched (30–100 times mean CI values) and display a slight increase from LREE to HREE. Thus, relative to the mean CI concentrations of REE, clear and significant (30–300 fold) enrichments in REE concentration are observed for the three apatite grains, which is in agreement with REE results for other Ryugu samples that found apatite to be the main REE bearing phase (Nakamura et al. 2022a) and are, furthermore, in line with the research by Morlok et al*. (*Morlok et al. 2006*)*. A direct comparison of the scale of the REE concentrations reported for other Ca-phosphates in the CI carbonaceous chondrite Orgueil (Morlok et al. 2006) reveals that the REE abundances of apatite grain 2 of Ryugu fragment A0055 (A0055 Pt2) and grain 1 of fragment C0076 (C0076 Pt1), 80–100 times CI and 30–60 times CI, respectively, are not dissimilar in scale to those of Orgueil Ca-phosphates that range between ~ 30 and ~ 100 times CI values (Morlok et al. 2006), apatite grains in ordinary chondrites that range between ~ 10 and 100 times CI values (Zhang et al. 2016), and those of apatite in the ungrouped carbonaceous chondrite DaG 978 that range between ~ 4 and 60 times CI values (Zhang et al. 2016). The REE enrichments of apatite grain 1 of Ryugu fragment A0055 (A0055 Pt1), roughly 250–300 times greater than the mean CI REE concentrations, are not only noticeably higher than the REE concentrations of the other two apatite grains in Ryugu but also much greater than the REE concentrations of the Ca-phosphate grains in Orgueil (Morlok et al. 2006). Similarly, high LREE concentrations have been reported in grains or merrillite, an anhydrous Mg–Na-bearing Ca-phosphate, in the ungrouped chondrite DaG 978 with concentrations of 150–300 times mean CI values for REEs up to Eu (Zhang et al. 2016).

In contrast to the three apatite grains, the dolomite grain (A0055 Pt 3) reveals a weaker enrichment of light to medium REE, of approximately 4–8 mean CI concentrations (Fig. 6). The concentration levels of REEs heavier than Dy (e.g., Ho, Er, Yb) were below the detection limits in the dolomite grain. This is in agreement with LREE enrichments observed in dolomite grains of other Ryugu samples (e.g., C0053, (Nakamura et al. 2022a)). All four matrix points of the Ryugu fragments (A0055 Pt4 and C0076 Pt2, Pt3 and Pt4) reveal a slight increase from LREE to HREE, but the two Ryugu fragments appear to differ with regard to their matrix Eu concentrations. Unlike the A0055 matrix (A0055 Pt4) that displays a noticeable negative Eu anomaly, C0076 matrix point 4 shows a clear positive Eu anomaly, the latter also appearing to be the case for the other two matrix points of C0076 (Pts 2 and 3), although admittedly the large error bars of Pt2 and Pt3 call for observational caution in this case.

The REE-concentrations of two of the matrix measurements in Ryugu sample C0076 (C0076 Pt2 and Pt3) are slightly depleted relative to the mean CI concentrations and are, thus, similar to the REE abundances in the matrices of CI chondrites, which are usually close to or depleted compared to bulk CI values (Morlok et al. 2006). This supports the recent classification of Ryugu material as CI chondrite following bulk XRF analysis, bulk isotopic ICP–MS analyses, (Yokoyama et al. 2022) and recent muonic X-ray results of 10 coarse Ryugu samples that have recorded major elemental abundances and isotopic compositions of Ryugu similar to those of CI chondrites (Nakamura et al. 2022b). Interesting, however, are the REE concentrations for A0055 matrix Pt4 and C0076 matrix Pt4 which show a noticeable (0–fivefold on average) enrichment relative to the CI mean REE concentrations. A similar observation was made by Yokoyama et al*.* (Yokoyama et al. 2022) during the sample bulk analyses.

Apart from the different directions of the Eu anomalies in the two matrices, a comparison of the REE quantification results for Ryugu fragment A0055 with those of fragment C0076 reveals a further difference between the two samples. Whereas the slight depletion in C0076 matrix points Pt2 and Pt3 fit well against the contrasting REE-enrichment of C0076 apatite grain Pt1 this is not the case for A0055, where both noticeable and significant REE enrichments are recorded for the matrix (A0055 Pt4) and the two apatite grains (A0055 Pt1 and Pt2), respectively. One explanation, although unlikely, for the REE enrichment of matrix point A0055 Pt4 (and/or C0076 Pt4) might be very small and unnoticed REE enriched apatite grains or grain fragments along the path of the primary beam indicated in Fig. 5, as well as on the path toward the XRF detector, that contribute their REE fluorescence to the total spectrum of the matrix.

## Conclusions

High energy X-ray fluorescence spectroscopy experiments were performed at beamline ID15A of the ESRF (Grenoble, France) on two rock fragments (A0055 and C0076) returned by the Hayabusa2 mission of JAXA. The sub-micron sized excitation beam of 90 keV allowed for the non-invasive investigation of these millimetre-sized samples, and extracting information on the rare earth element composition from the entire sample volume.

Several points of interest were identified based on the elemental distribution images obtained from two-dimensional overview scans. Local XRF analyses combined with an absorption–CT-aided and reference-material-based fundamental parameter quantification method was used to derive quantitative REE information from the identified points of interest. Combined with the XRF data, computed tomography virtual slices were utilised to determine the path length distances within the intersected phases and to identify and measure the grains of interest along the primary beam path to be able to provide local REE concentration values within the grains, contained within the millimetre-sized rock fragments.

It was found that REEs are mainly enriched within certain Ca- and Sr-rich phases, which are suggested to be apatite following the research by Morlok et al. 2006 and Zhang et al. 2016. Other REE-poorer but Mn-enriched Ca phases are identified as likely to be dolomite. The obtained REE enrichment trends are also compared to a location attributed to the general A0055 matrix (i.e*.*, no significant carbonate or high density phases were identified in this location). REE enrichment factors of several orders of magnitude compared to the mean CI chondrite composition were found for the apatite grains within the A0055 and C0076 Ryugu rock fragments, whereas only an approximately factor 10 enrichment for LREE up to Dy was found for the dolomite phase and slightly above 1 for the matrix phases.

The proposed method is shown to be a powerful tool in the preliminary analysis of millimetre-sized asteroidal rock fragments. Not only does the method provide fast identification of microscopic grains of interest within the large sample bulk, providing relative coordinates that can be used for further investigation of said detected grains by other analysis methods. The method at hand also delivers semi-quantitative information on the relative abundance of REEs with respect to the mean CI chondritic composition. The flat but highly enriched REE pattern of apatite grain 1 in Ryugu rock fragment A0055 indicates that this grain did not experience any fractionation during aqueous alteration on the Ryugu asteroid. The other two apatite grains show a bit lower enrichment with a slight increase from LREE to HREE, a pattern which is quite similar to several Orgueil apatite grains studied by Morlock et al. (Morlok et al. 2006). Matrix values are slightly enriched for Ryugu rock fragment A0055 and point 2 of rock fragment C0076 but depleted for points 3 and 4. The unusual high enrichment of both apatite grain 1 and matrix in rock fragment A0055 suggest a much higher alteration than in rock fragment C0076, which underlines the inhomogeneous and brecciated nature of Ryugu.

## Supplementary Information

**Additional file1: Figure S1** XRF spectra corresponding to the measurements in points 1 to 4 in rock fragment C0076 (600 s/pt). XRF spectra were normalised for the Ta–K_α_ signal intensity to provide more straightforward comparison. Magnified inserts of two select energy ranges marked by dashed bounding boxes in light blue and red are displayed in parts B and C, respectively. **Figure S2** CT slices that were obtained at SPring-8 beamline 20X (Nakamura et al. 2022b) show the positions of the points of interest indicated in Figure A1 in Ryugu rock fragment C0076. A red arrow marks the primary X-ray beam path and direction, fluorescence detector was positioned at the left of the CT image. Yellow circles indicate the Ca-rich grains (point 1) from which REE information is primarily obtained. Points 2–4 are matrix measurements and as such have no distinct Ca-rich grains. Separate grains along the beam path are indicated by their respective size along the beam path in orange, along with an estimate of the mineralogical phase for the larger grains.

## Data Availability

Data are available from the authors upon reasonable request.

## Abbreviations

CT: Computed tomography
ESRF: The European synchrotron radiation facility
HE: High energy
HREE: High (atomic number) REEs
ICP–MS: Inductively coupled plasma mass spectrometry
JAXA: Japanese aerospace exploration agency
LREE: Low (atomic number) REEs
REE: Rare earth element
SR: Synchrotron radiation
XRF: X-ray fluorescence
