# Supplementary material for: Rare earth element identification and quantification in millimetre-sized Ryugu rock fragments from the Hayabusa2 space mission
Source: Earth Planets Space. 2022 Sep 28;74(1):146. doi: 10.1186/s40623-022-01705-3 (PMC9516535; doi:10.1186/s40623-022-01705-3)
Supplement: Supplementary file 1 — Additional file1: Figure S1 XRF spectra corresponding to the measurements in points 1 to 4 in rock fragment C0076 (600 s/pt). XRF spectra were normalised for the Ta–Kα signal intensity to provide more straightforward comparison. Magnified inserts of two select energy ranges marked by dashed bounding boxes in light blue and red are displayed in parts B and C, respectively. Figure S2 CT slices that were obtained at SPring-8 beamline 20X (Nakamura et al. 2022b) show the positions of the points of interest indicated in Figure A1 in Ryugu rock fragment C0076. A red arrow marks the primary X-ray beam path and direction, fluorescence detector was positioned at the left of the CT image. Yellow circles indicate the Ca-rich grains (point 1) from which REE information is primarily obtained. Points 2–4 are matrix measurements and as such have no distinct Ca-rich grains. Separate grains along the beam path are indicated by their respective size along the beam path in orange, along with an estimate of the mineralogical phase for the larger grains. [file 40623_2022_1705_MOESM1_ESM.pdf]

Rare Earth Element identification and quantification in millimetre-sized Ryugu rock fragments from the Hayabusa2 space mission – Additional File

Author #1: **Pieter Tack, Ghent University, Dept. of Chemistry, XMI, Krijgslaan 281 S12, 9000 Ghent, Belgium, pieter.tack@ugent.be**
Author #2: Ella De Pauw, Ghent University, Dept. of Chemistry, XMI, Krijgslaan 281 S12, 9000 Ghent, Belgium, ella.depauw@ugent.be
Author #3: Beverley Tkalcec, Goethe University, Dept. of Geoscience, Altenhoeferallee 1, 60438 Frankfurt, Germany, tkalcec@em.uni-frankfurt.de
Author #4: Miles Lindner, Goethe University, Dept. of Geoscience, Altenhoeferallee 1, 60438 Frankfurt, Germany, mileslindner@googlemail.com
Author #5: Benjamin Bazi, Ghent University, Dept. of Chemistry, XMI, Krijgslaan 281 S12, 9000 Ghent, Belgium, benjamin.bazi@ugent.be
Author #6: Bart Vekemans, Ghent University, Dept. of Chemistry, XMI, Krijgslaan 281 S12, 9000 Ghent, Belgium, bart.vekemans@ugent.be
Author #7: Frank Brenker, Goethe University, Dept. of Geoscience, Altenhoeferallee 1, 60438 Frankfurt, Germany and IHGP, University of Hawaii, Menoa, f.brenker@em.uni-frankfurt.de
Author #8: Marco Di Michiel, The European Synchrotron, ESRF, 38000 Grenoble, France, dimichie@esrf.fr
Author #9: Masayuki Uesugi, JASRI/SPring-8, Sayo, 679-5198, Japan, uesugi@spring8.or.jp
Author #10: Hisayoshi Yurimoto, Hokkaido University, Sapporo 060-0810, Japan, yuri@ep.sci.hokudai.ac.jp
Author #11: Tomoki Nakamura, Tohoku University, Sendai 980-8578, Japan, tomoki.nakamura.a8@tohoku.ac.jp
Author #12: Kana Amano, Tohoku University, Sendai 980-8578, Japan, amakana@dc.tohoku.ac.jp
Author #13: Megumi Matsumoto, Department of Earth Science, Tohoku University; Aoba-ku, Sendai, 980-8578, Japan, m_matsumoto@tohoku.ac.jp
Author #14: Yuri Fujioka, Department of Earth Science, Tohoku University; Aoba-ku, Sendai, 980-8578, Japan, yuri.fujioka.r1@dc.tohoku.ac.jp
Author #15: Yuma Enokido, Department of Earth Science, Tohoku University; Aoba-ku, Sendai, 980-8578, Japan, yuma.enokido.r8@dc.tohoku.ac.jp
Author #16: Daisuke Nakashima, Department of Earth Science, Tohoku University; Aoba-ku, Sendai, 980-8578, Japan, dnaka@tohoku.ac.jp
Author #17: Takaaki Noguchi, Kyoto University, Kyoto 606-8502, Japan, noguchi.takaaki.2i@kyoto-u.ac.jp
Author #18: Ryuji Okazaki, Kyushu University, Fukuoka 812-8581, Japan, okazaki.ryuji.703@m.kyushu-u.ac.jp
Author #19: Hikaru Yabuta, Hiroshima University, Higashi-Hiroshima 739-8526, Japan, hyabuta@hiroshima-u.ac.jp
Author #20: Hiroshi Naraoka, Kyushu University, Fukuoka 812-8581, Japan, naraoka.hiroshi.885@m.kyushu-u.ac.jp
Author #21: Kanako Sakamoto, ISAS/JAXA, Sagamihara 252-5210, Japan, sakamoto@planeta.sci.isas.jaxa.jp
Author #22: Shogo Tachibana, The University of Tokyo, Tokyo 113-0033, Japan and ISAS/JAXA, Sagamihara 252-5210, Japan, tachi@eps.s.u-tokyo.ac.jp

Author #23: Toru Yada, Institute of Space and Astronautical Science (ISAS), Japan Aerospace Exploration Agency (JAXA), Sagamihara 252-5210, Japan, yada@planeta.sci.isas.jaxa.jp
Author #24: Masahiro Nishimura, Institute of Space and Astronautical Science (ISAS), Japan Aerospace Exploration Agency (JAXA), Sagamihara 252-5210, Japan, nishimura.masahiro2@jaxa.jp
Author #25: Aiko Nakato, Institute of Space and Astronautical Science (ISAS), Japan and Aerospace Exploration Agency (JAXA), Sagamihara 252-5210, Japan, nakatoh.aiko@jaxa.jp
Author #26: Akiko Miyazaki, Institute of Space and Astronautical Science (ISAS), Japan and Aerospace Exploration Agency (JAXA), Sagamihara 252-5210, Japan, miyazaki@planeta.sci.isas.jaxa.jp
Author #27: Kasumi Yogata, Institute of Space and Astronautical Science (ISAS), Japan and Aerospace Exploration Agency (JAXA), Sagamihara 252-5210, Japan, yogata.kasumi@jaxa.jp
Author #28: Masanao Abe, Institute of Space and Astronautical Science (ISAS), Japan and Aerospace Exploration Agency (JAXA), Sagamihara 252-5210, Japan, abe@planeta.sci.isas.jaxa.jp
Author #29: Tatsuaki Okada, Institute of Space and Astronautical Science (ISAS), Japan and Aerospace Exploration Agency (JAXA), Sagamihara 252-5210, Japan, okada@planeta.sci.isas.jaxa.jp
Author #30: Tomohiro Usui, Institute of Space and Astronautical Science (ISAS), Japan and Aerospace Exploration Agency (JAXA), Sagamihara 252-5210, Japan, usui.tomohiro@jaxa.jp
Author #31: Makoto Yoshikawa, Institute of Space and Astronautical Science (ISAS), Japan and Aerospace Exploration Agency (JAXA), Sagamihara 252-5210, Japan, yoshikawa.makoto@jaxa.jp
Author #32: Takanao Saiki, Institute of Space and Astronautical Science (ISAS), Japan and Aerospace Exploration Agency (JAXA), Sagamihara 252-5210, Japan, saiki.takanao@jaxa.jp
Author #33: Satoshi Tanaka, Institute of Space and Astronautical Science (ISAS), Japan and Aerospace Exploration Agency (JAXA), Sagamihara 252-5210, Japan, tanaka@planeta.sci.isas.jaxa.jp
Author #34: Fuyuto Terui, Kanagawa Institute of Technology, Atsugi 243-0292, Japan, t5369@cce.kanagawa-it.ac.jp
Author #35: Satoru Nakazawa, Institute of Space and Astronautical Science (ISAS), Japan Aerospace Exploration Agency (JAXA), Sagamihara 252-5210, Japan, nakazawa.satoru@jaxa.jp
Author #36: Sei-Ichiro Watanabe, Nagoya University, Nagoya 464-8601, Japan, seicoro@eps.nagoya-u.ac.jp
Author #37: Yuichi Tsuda, ISAS/JAXA, Sagamihara 252-5210, Japan, tsuda.yuichi@jaxa.jp
Author #38: Laszlo Vincze, Ghent University, Dept. of Chemistry, XMI, Krijgslaan 281 S12, 9000 Ghent, Belgium, laszlo.vincze@ugent.be

Figure A1:

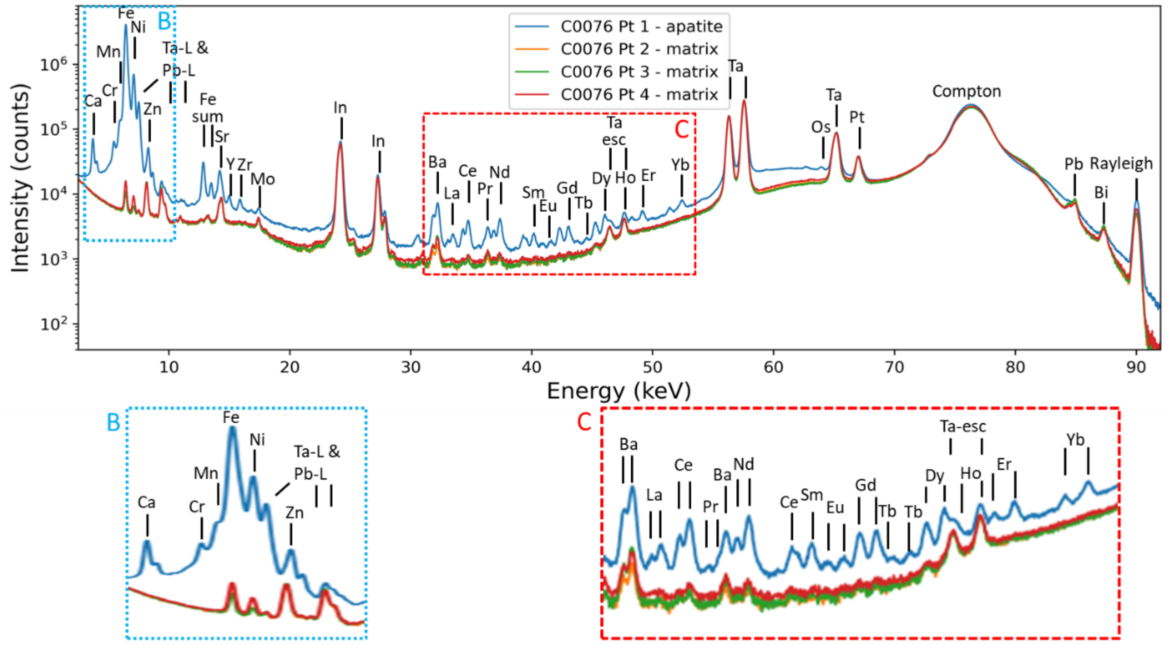

Figure A1: XRF spectra corresponding to the measurements in points 1 to 4 in rock fragment C0076 (600 s/pt). XRF spectra were normalised for the Ta-K$_{\alpha}$ signal intensity to provide more straightforward comparison. Magnified inserts of two select energy ranges marked by dashed bounding boxes in light blue and red are displayed in parts B and C, respectively.

Figure A2:

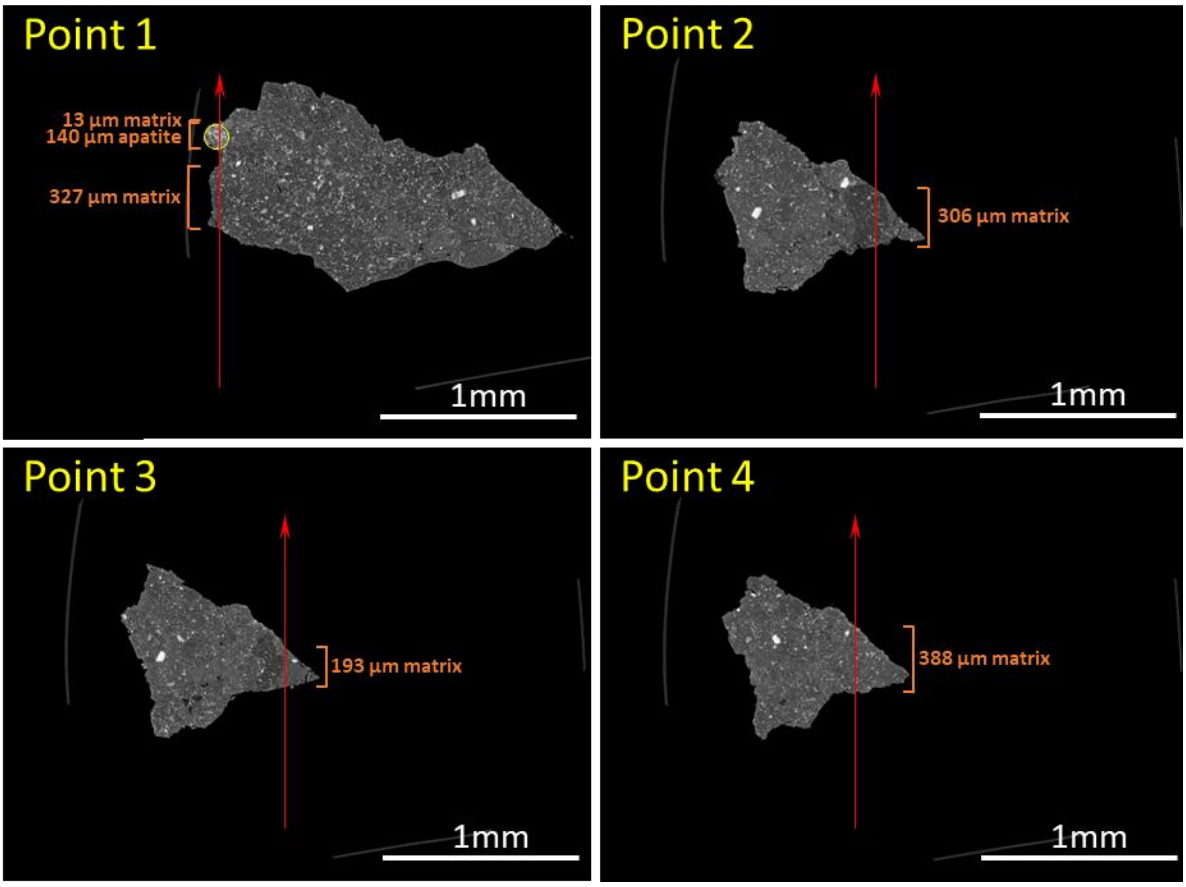

Figure A2: CT slices that were obtained at SPring-8 beamline 20XU (Nakamura et al., 2022b) show the positions of the points of interest indicated in Figure A1 in Ryugu rock fragment C0076. A red arrow marks the primary X-ray beam path and direction, fluorescence detector was positioned at the left of the CT image. Yellow circles indicate the Ca-rich grains (point 1) from which REE information is primarily obtained. Points 2-4 are matrix measurements and as such have no distinct Ca-rich grains. Separate grains along the beam path are indicated by their respective size along the beam path in orange, along with an estimate of the mineralogical phase for the larger grains.
